# The Impact of Hospital Pharmacy Operation on the Quality of Patient Care

**DOI:** 10.3390/ijerph20054137

**Published:** 2023-02-25

**Authors:** Karolina Wylegała, Urszula Religioni, Marcin Czech

**Affiliations:** 1 Chair and Department of Pharmacoeconomics and Social Pharmacy, Medical University of Poznan, 61-701 Poznan, Poland; 2 Independent Public Healthcare Center in Miedzychod, 64-400 Miedzychod, Poland; 3 School of Public Health, Centre of Postgraduate Medical Education of Warsaw, 01-813 Warsaw, Poland; 4 Department of Pharmacoeconomics, The Institute of Mother and Child, 01-211 Warsaw, Poland; 5 Business School, Warsaw University of Technology, 00-661 Warsaw, Poland

**Keywords:** hospital pharmacy, drug management, quality of care, unit-dose, multi-dose

## Abstract

This paper presents the role, tasks, and place of a hospital pharmacy in the structure of the entire facility. The role of hospital drug management and pharmacy seems to be extremely important in providing patients with high-quality care. Particular emphasis was placed on the distribution systems of medicinal products and medical devices in the hospital. The advantages and disadvantages of the classical distribution system and modern systems such as unit-dose and multi-dose—and the most important differences between them—are presented. Difficulties related to implementing modern distribution systems in hospitals were also discussed. The information provided is presented in the context of the legal regulations in Poland.

## 1. Introduction

Pursuant to Art. 86, Point 1, of the Pharmaceutical Law Act of September 6, 2001, a pharmacy is a public health facility where authorized persons provide pharmaceutical services [1]. Actually, this general definition captures a wide range of activities, including, among others, dispensing medicinal products and medical devices, supplying hospital organizational units with medicinal products and medical devices, preparing prescription drugs, providing information on medicinal products and medical devices, organizing the supply of medicinal products and medical devices to the hospital, participating in the monitoring of adverse drug reactions, conducting clinical trials in the hospital, rationalizing pharmacotherapy, and managing medicinal products and medical devices. Hospital pharmacy employees increasingly often participate in making important therapeutic decisions, for example, by playing an important role in hospital formula preparation or therapeutic committee functions and making decisions about the use of specific, often very expensive therapies.

According to the data of the Central Register of Pharmacies from September 9, 2022, there are 565 hospital pharmacies and 1035 hospital pharmacy departments in Polish healthcare entities [2]. According to these data, most hospitals have hospital pharmacy departments, not hospital pharmacies, but for the purposes of this study, the term "hospital pharmacy" refers to both of these forms, as the aim of the study is not to show differences in their functioning.

The role of the hospital pharmacy in controlling the operating costs of a hospital facility cannot be overestimated. Due to the general progress in medicine, in particular in the field of pharmacotherapy, especially in such therapeutic areas as oncology, hematology, rheumatology, or rare diseases, and other factors such as the demographic and epidemiological situation, health expenditure, including costs directly related to treatment, is increasing in many countries [3]. This is also the case in Poland, where public expenditure on medical products (including medicines) alone amounts to 22.7% of total healthcare expenditure by function [4].

Proper management of pharmacotherapy is one of the key elements of managing a hospital, which should guarantee the highest possible level of safety of the services provided, clinical effectiveness of the therapy, and rationalization of the funds allocated for this purpose [5,6]. The proper functioning of the hospital, including rational drug management, is a task for human teams that cooperate on many levels and are managed by responsible persons under the supervision of the founding bodies. In view of the above, proper communication and joint work of the entire medical team are of great importance for both the safe and rational treatment of hospitalized patients, and the hospital pharmacy, with its staff and entire infrastructure, has one of the most important roles to play in this network.

Therefore, the aim of this article is to present a general outline of the functioning of a hospital pharmacy, its tasks and scope of responsibility, as well as the increasingly broader role of pharmacists in the entire therapy process. A particular goal is to present modern drug distribution systems in hospitals and demonstrate the extent to which they affect the functioning of a given unit and the level of patient care.

The topic raised is extremely important due to the large differences in the organization and functioning of hospital pharmacies around the world. Despite the high level of technological advancement and automation of many processes in hospital pharmacies, e.g., in Western European countries, some countries, including Poland, still rely on classic methods of hospital drug distribution. The low technological advancement of hospitals may lead both to a lack of optimization of the quality of patients’ therapy and a lack of cost rationalization.

## 2. The Role and Place of the Pharmacy within Hospital Structure

A pharmacy operating in a modern hospital fulfills many real tasks related to the medical activity of a given institution. It is directly or indirectly related to all entities involved in the production or distribution of medicinal products, and the Pharmaceutical Inspection exercises control over the statutory activities of the pharmacy. The obligation to establish a hospital pharmacy in each facility that is a hospital or other medical entity that performs stationary and 24-h health services results from Art. 87 of the Pharmaceutical Law [1].

The primary role of a hospital pharmacy is to supply drugs, although practically the entire process of distribution of medicinal products falls within the competence of the hospital pharmacy, including the very important function of controlling, correcting, and informing patients about the medicinal products used.

The tasks of a hospital pharmacy include dispensing medicinal products and medical devices specified in separate regulations, preparing prescription drugs and pharmacy drugs, providing information on medicinal products and medical devices, supplying hospitals with medicinal products and medical devices, preparing medicines for enteral and parenteral nutrition, preparing drugs in daily doses, including cytostatic drugs, preparing radiopharmaceuticals, preparing infusion fluids, preparing solutions for hemodialysis and intraperitoneal dialysis, monitoring adverse effects of medicinal products, participation in clinical trials conducted in the hospital, and supplying hospitals with medicinal products and medical devices [5,6,7].

Pursuant to Article 86, Point 4, of the Pharmaceutical Law Act, the hospital pharmacy develops procedures for dispensing drugs and medical devices to specialist hospital departments. In addition, the hospital pharmacy keeps records of samples for clinical trials and those obtained from donations of medicinal products and medical devices. 

The tasks of each hospital pharmacy also include keeping track of the messages of the Chief Pharmaceutical Inspector on the suspension or withdrawal of a medicinal product from the market, securing suspended or recalled batches of drugs in the pharmacy and at departments, keeping records of reports on precautionary measures taken, drug disposal overdue in the hospital pharmacy and hospital departments, monitoring the stock level at a level that guarantees meeting the current needs of departments and proper functioning in emergency situations, and supervision of the conditions of storage of drugs and medical devices in properly secured conditions in each unit supplying the aforementioned products. A hospital pharmacy may also supply drugs to other entities performing medical activities, e.g., inpatient facilities providing 24-h health services or outpatient health services.

One of the dynamically developing tasks of a hospital pharmacy is the preparation of drugs under aseptic conditions, e.g., cytotoxic drugs, dietary drugs, antibiotics, and infusion fluids. Appropriate activities in this area significantly affect the quality of pharmacotherapy for patients while minimizing the number of errors related to the preparation of these drugs [8,9]. This area of the hospital pharmacy’s activity is associated with the obligation to ensure the special quality of parenteral drugs in accordance with the principles of good manufacturing practice (GMP) and taking patient safety into account. The use of solutions ensuring a clean environment has become a necessity resulting from GMP, e.g., air filtration systems, maintaining a cascade pressure difference between rooms, microbiological cleanliness, physical cleanliness, and in relation to hazardous drugs—also environmental decontamination and employee protection. This necessitates the use of quality assurance systems to ensure an aseptic environment that is monitored and validated.

As mentioned before, drug management is largely related to the financial aspect. Therefore, it is not surprising that apart from the tasks strictly related to the profession of pharmacist, within the meaning of the relevant acts, the pharmacy’s activity must comply with such legal acts as the Public Procurement Law Act in relation to tenders or the Public Finance Act in relation to expenditure rationalization and, more broadly, budget discipline.

## 3. The Role of a Pharmacist in the Hospital Drug Management System

A hospital pharmacist’s primary responsibility should be to ensure that patients’ pharmacotherapy is safe and effective. As in the case of the definition of a hospital pharmacy quoted at the beginning, this general term includes a number of detailed activities that, apart from the tasks related to the treatment itself, also include obligations regarding the provision of appropriate technical conditions, drug trade, their purchase, and supervision of first aid kits at individual departments, participation in clinical trials conducted in a hospital and in drug research, monitoring of adverse effects of medicinal products, and providing this information to the competent authorities, as well as providing pharmaceutical services that are performed only in the hospital pharmacy, e.g., cytotoxic drugs or enteral and parenteral nutrition [10,11,12]. In practice, the work of a pharmacist employed by a medical entity, e.g., a hospital, is adapted to the specifics of the facility and the profile of patients treated there. For this reason, medical universities in Poland allow pharmacists to specialize in the field of, among others: clinical, hospital, or pharmacy [13].

In a modern hospital, the role of a pharmacist goes beyond this brief presentation of the basic scope of tasks. A clinical or hospital pharmacist should collaborate directly with a physician ordering pharmacotherapy for hospitalized patients in order to select the drug substance more effectively and safely, especially in patients undergoing polytherapy. A pharmacist should be a physician advisor and educator for patients and hospital medical staff. The tasks that a pharmacist should perform include informing patients and healthcare professionals about the effects of a drug, observing the occurrence of side effects, anticipating the effects of drug interactions with food and drug administration, preventing dosing and administration errors, including polypragmasy, and supervising drug management in hospital departments [14]. 

Education provided by a hospital pharmacist should be a standard, covering both patients and nursing staff regarding taking medications, possible side effects, and many other topics in order to eliminate errors in the preparation, storage, frequency of dosing, or administration of drugs [15,16,17].

It is extremely important for hospital pharmacists to participate in the Therapeutic Committee [18]. The tasks of the Therapeutic Committee include: creating and updating a hospital formula; determining procedures for drug management and pharmacotherapy; developing and assessing therapy standards for selected disease entities; setting standards of conduct in specific clinical cases in accordance with the current recommendations of scientific societies; determining the principles of rational drug management; creating a hospital list of drugs with a division into their availability categories; analyzing applications for the introduction and deletion of a drug from the prescription; educating other medical employees of the hospital on the implementation and functioning of procedures related to pharmacotherapy in the hospital; and analyzing the consumption of drugs and medical devices by individual hospital departments. The Therapeutic Committee should collaborate with other teams in the facility, including the Quality, Antibiotic Treatment, and Nosocomial Infections Committees [19].

As already mentioned, one of the main tasks of the Therapeutic Committee is to create and update the hospital formula, which is a list of drugs that should be followed by doctors when making therapeutic decisions. The hospital formula should be adapted to the profile of the medical entity and define all the rules of drug management in the facility, from the moment of purchasing medicinal products by the hospital pharmacy to administering them to the patient. The priority in creating a hospital formula should be the maximization of therapeutic benefits while maintaining a favorable financial balance [6,19].

## 4. Technical and Organizational Requirements for a Hospital Pharmacy

The regulations governing the premises, technical, and organizational conditions of the operation of a hospital pharmacy do not clearly define the requirements to be met by the premises of the pharmacy department and the staff employed therein [20,21]. The organization and equipment of a hospital pharmacy therefore depend on the scope of pharmaceutical services provided in a given facility, including, e.g., preparation of prescription drugs, preparation of cytotoxic drugs, parenteral and enteral nutrition, and infusion fluids [22]. 

In the case of preparation of drugs for parenteral nutrition, enteral nutrition, or cytotoxic drugs, the base area should be enlarged proportionally to the type of services provided. In a situation where the hospital pharmacy will produce modified infusion fluids, the base area should be enlarged, and additionally, a quality control laboratory should be established with the possibility of conducting physicochemical and microbiological tests. However, there is a flexible alternative, requiring the written consent of the pharmaceutical inspector, to outsource control tests. In the case that the pharmacy prepares cytotoxic drugs, it is absolutely necessary to comply with the principles of occupational health and safety specified in separate regulations [20].

Depending on the type of pharmaceutical services provided in a given facility, tailored to its profile, a hospital pharmacy may meet the statutory minimum or include additional specific laboratories.

## 5. Classic Methods of Drug Distribution in a Hospital

In every hospital, the statutory obligations of a hospital pharmacy should be described in the procedures in accordance with the provisions of the Pharmaceutical Law Act and other documents, e.g., the Regulation of the Minister of Health on Good Distribution Practice [23] or WHO Operational Principles for Good Pharmaceutical Procurement [24].

The hospital pharmacy organizes the supply of medicinal products and monitors the entire purchasing process of medicinal products and medical devices on an ongoing basis. The head of a hospital pharmacy participates in all stages related to the supply of a medical facility with an appropriate range of medicinal products and medical devices, in accordance with the hospital formula. This applies to cost planning, conducting tenders, and broadly understood cooperation with specialized external companies, aimed at ensuring quantitative and product flexibility [6].

The hospital pharmacy also participates in the distribution of drugs in the hospital in close cooperation with the heads of individual departments, ensuring a good and effective flow of information. 

In the traditional model of drug distribution in a hospital, a pharmacy only fulfills the collective drug requirements for individual organizational units, with no control over the amount of drugs actually administered to patients. Medicines dispensed from the central pharmacy to the departmental first-aid kits are recorded in the accounts of the pharmacy and the given department, but the further fate of the drug is not visible to the pharmacy employees. In addition, in many facilities in Poland, orders are handwritten by doctors, bypassing the computer system. 

The recommended method of drug delivery is the "just-in-time" (JIT) model, which is a management technique where all the necessary products (in this case, medicinal products and medical devices) are delivered at the required time and in the required quantity. The main benefit is that lead times are reduced to a minimum, which brings significant savings in inventory reduction [6]. 

In the majority of Polish hospitals, drug management is based on the so-called departmental first aid kits, i.e., the storage of medicines and medical devices in a properly separated and secured place in the department of medicines and medical devices collected from the central pharmacy on the basis of demand. After the hospital pharmacy releases medicines to the departments, the pharmacy controls the correctness of their storage and protection against unauthorized persons. Department nurses and department heads are responsible for storing and securing medicines in hospital wards. 

As a rule, in hospitals in Poland, drugs, medical devices, and disinfectants ready for use are delivered to the wards by internal transport and issued from the pharmacy on the basis of delivered prescriptions signed by the head of the clinic or a doctor authorized by him, managers of laboratories and departments, or the doctors on duty. Psychotropic and narcotic drugs are dispensed from a pharmacy on the basis of prescriptions signed by doctors responsible for the management of these drugs in a given department. If the prescribed medication is not available or it is not possible to carry it out, the manager of the pharmacy communicates with the doctor who prescribed the medication and indicates substitute medications or a different method of preparing the medication. If a drug is essential and cannot be replaced by another drug, the hospital pharmacy will make every effort to purchase the correct drug. The pharmacy also archives copies of prescriptions and completed orders in its files for five years and maintains appropriate billing and reporting documentation [24].

An emergency ordering procedure is very important from the point of view of securing patients’ access to drugs. The rules of this procedure must be known to all medical staff in the hospital, and the procedure must have specific persons responsible for each stage. Some hospitals create internal regulations for the so-called inter-ward first aid kit, in which the so-called emergency medications are used only in emergency cases of threat to the patient’s life or health [6].

All medicinal products, both in the central pharmacy and in departmental pharmacies, must be stored in properly validated conditions, in accordance with the Regulation of the Minister of Health on the basic conditions for running a pharmacy, i.e., in a manner that guarantees compliance with the quality requirements and safety of storage established for a medicinal product or medical device and under the conditions specified in the safety data sheet. The parameters that should be monitored on an ongoing basis and recorded in the internal documents of the entity are temperature and air humidity. For this purpose, some establishments use an electronic system for monitoring temperature and humidity values in designated pharmacy rooms and refrigerators [6,22,23].

In the majority of Polish hospitals, pharmacies work only in a one-shift system, which is associated with the need to collect the necessary supply of drugs in hospital wards. It is very difficult to define what the minimum stock of medicinal products should be. It is difficult to predict which medicinal products or medical devices will be needed on a given day in specific departments, how many new patients will be admitted, how the condition of already hospitalized patients will change, and whether the patient will experience side effects related to the use of certain products. Despite the divergent treatment profiles in individual wards, the same drugs are usually stored in each ward. Each hospital’s ward first aid kit is estimated to be worth several thousand zlotys, while hospital pharmacies discard several to several kilograms of overdue drugs that are unused at the ward level each year. Disposal costs are constantly increasing because there are a large number of equivalents on the market and patients use different doses of drugs and preparations [25,26,27].

Taking into account the control of drug storage conditions and their protection, in hospitals with classic ward first aid kits, it is recommended that pharmacists carry out routine checks, including storage conditions for drugs, including psychotropic and potent drugs [28,29].

## 6. Modern Methods of Drug Distribution in the Hospital

**Figure 1:**
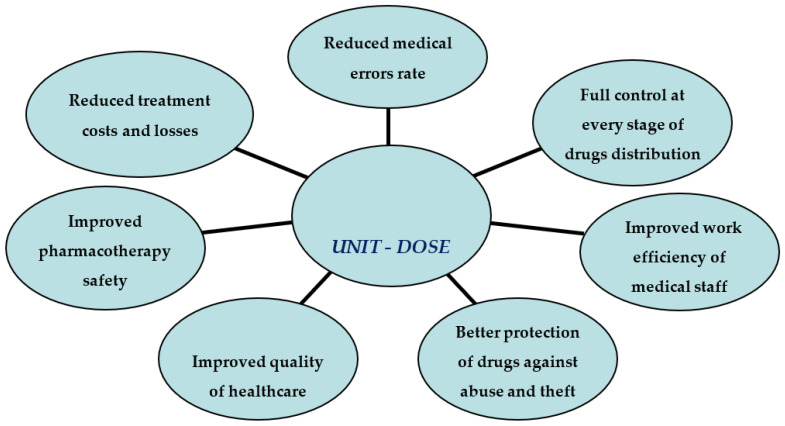
The advantages of implementing a unit-dose system.

Due to the increasing demand for pharmacists, the extensive duties of nurses working in departments, and the ever-increasing costs of pharmacotherapy, medical entities are looking for modern solutions to improve and control drug management. This necessity also results from the need to ensure patient safety more closely, including reducing medication errors, improving the quality of care for hospitalized patients, and thus meeting the standards of service provision by hospital pharmacies. An automated unit-dose distribution system (Figure 1) may be a good solution. The main assumption of the unit-dose system is the mechanical preparation and distribution of drugs in a ready-to-administer and individually prepared form for a given patient from a central pharmacy [30,31].

Distributing medicinal products in the form of individual doses allows one to maintain control at every stage of distribution, from the purchase of the drug to its administration to the patient. The unit-dose system limits the dispensing of drugs outside the hospital and the patients taking their drugs in the hospital, thus contributing to the reduction of losses caused by poor drug storage, exceeding their expiry dates, and thus the disposal of unused drugs from departmental first aid kits [29]. The system is designed to ensure a pharmacist-controlled process of delivering drugs prescribed on an electronic prescription to the department in individual doses. Medicines packed by a machine in a hospital pharmacy are ready to be administered to a specific patient without the need to prepare them in the ward by nurses, and identification is often done using barcodes. The unit-dose system only allows the storage of emergency drugs and drugs routinely used in a given department in the amounts determined by internal procedures in departmental first aid kits. In addition, the functioning of the individual dose system allows for the definition of rules for the return of unused funds (e.g., due to the changing of a patient’s clinical situation), which prevents wasting single doses of drugs [30]. 

The operation of a unit-dose system prepared in a hospital pharmacy for a specific patient involves two key elements:

1. Pharmacist reviewing all prescriptions in terms of content and potential drug interactions as well as prescribed doses;
2. Separation of drugs into unit doses, each of which is packaged separately and has an attached label with the name of the drug, active substance content, serial number, and expiry date [32].

In hospital pharmacies preparing drugs in the unit-dose system, there must be properly prepared rooms for preparing drugs in daily doses, including a separate place for machines separating blisters and packing drugs into individual packages, as well as a separate administrative area with a station for analyzing medical orders. The functioning of the unit dose is fully computerized, while the rules of communication between the hospital ward and the pharmacy are carried out using an e-prescription. It allows saving up to 25% of the pharmacist’s working time [33]. There are also systems in which drug orders are entered into a computer directly by doctors, which can shorten the communication chain and reduce the possibility of errors [34]. 

One of the greatest advantages of using the unit-dose system in hospitals is the reduction of the risk of mistakes by nursing staff when administering medication. The drug distribution system prepared for a specific patient significantly reduces the error rate, including potentially harmful errors [35]. Many of the errors that the unit-dose system minimizes relate to drug administration. This is particularly important given the prevalence of dispensing errors, including dispensing the wrong strength and the wrong dosage form [36]. 

An integral part of the unit-dose system is the electronic prescription system. Fontan et al. have shown that the percentage of errors in the case of handwritten prescriptions is as high as 87.9%, while in the case of prescriptions using a computer system, it is "only" 10.6%, with a statistically significant difference [37]. 

Medication errors are associated not only with complications and prolonged hospital stays for patients but also increase the costs incurred by the healthcare provider. Medical personnel should strive to identify weak links in the drug distribution process in the hospital and develop mechanisms to prevent errors because, as research shows, improper drug distribution is the cause of many errors in pharmacotherapy. The implementation of an effective drug distribution system in a hospital seems to be a necessary step towards ensuring the safety and optimization of pharmacotherapy. This is particularly important, taking into account the role of pharmacists and hospital pharmacies in optimizing therapy costs [38].

Analyses of medical errors occurring during the preparation of drugs in the unit-dose system indicate that the error rate at this stage ranges from 2.5% to 10.9%, mainly due to the omission of a drug dose (30.2%), the dispensing of an incorrect dose (31.8%) [39], and omitting the drug packaging [40]. The most common errors at the stage of administering drugs in the unit-dose system were the wrong time of administration, the wrong dose, and the omission or repetition of the dose [37,40,41].

The introduction of the unit-dose system also encounters obstacles, which in Polish conditions include, among others limited financial possibilities, insufficient computerization of hospitals, the need to reorganize work, change habits and learn new procedures, the need for a hospital pharmacy in a 24/7 system, unavailability of multi-tablet packaging, limited possibilities of using the unit-dose system in the so-called rapid medical assistance, e.g., in the Hospital Emergency Department, where it is not possible to wait for the release of drugs from the hospital pharmacy to the ward, and in the case of drugs containing psychoactive substances, the management and distribution of which is governed by separate rules of pharmaceutical law, the need to change the work standards of pharmacists developed over many years, nurses, doctors and the obligation of additional staff training and the organization of new infrastructure [42].

The unit-dose model is currently the most widely used drug distribution system in hospitals worldwide [43,44]. In Poland, in 2014, the unit-dose system was introduced, among others, in all departments at the Dr. W. Bieganski Regional Specialist Hospital in Grudziądz, St. Barbara Hospital in Sosnowiec, the University Teaching Hospital in Wrocław, and in selected hospital departments at the Teaching Hospital of the Transfiguration of Jesus in Poznań [45,46,47,48]. The management of the hospital allocated 7.9 million PLN (1.65 million euros) for the system in Grudziądz, and according to estimates, the savings resulting from the reduction of overdue drugs in departmental first aid kits amount to 140 thousand PLN (30 thousand euros) annually [45]. However, it should be noted that the cost of purchasing the system may vary both from the size of the hospital, the number and type of drugs it will serve, and the scope of its functionality. A study conducted in 2016 by Żuk et al., assessing the functioning of the unit-dose system, confirmed the benefits of its implementation and the satisfaction of the majority of users [44].

The second, also innovative, system of drug distribution in medical facilities is the system known as multi-dose. This system is based on controlled doses of drugs being automatically issued to patients from a dispenser at a hospital ward [49]. It is a combination of high-class hardware and software that optimizes and monitors pharmacotherapy in medical facilities. 

The components of the multi-dose system may include:

- modular and scalable software that allows to audit all activities, from ordering to administering the drug to the patient, and to carry out analyzes of treatment costs;
- an automated hospital pharmacy warehouse that manages inventory, orders, and drug deliveries to hospital units;
- a robotic dispenser of drugs and medical materials stationed in hospital wards that records each packaging issue, guaranteeing proper protection of drugs against unauthorized persons, and provides detailed information on the collection of the drug (number of packages, delivery times, etc.);
- computerized medical e-cart replacing traditional nursing carts with access to medical orders [50].

The most important differences between the multi-dose system and the unit-dose system relate to the possibility of comprehensive drug management in the hospital, including in individual departments, through the multi-dose (ward-based) system, while in the second case it is practically a classic automatic pharmacy system [49,51]. Other differences include: technical issues (software, integration, system installation location), management, efficiency in terms of the number of patients and drugs served, safety, hygiene, and, of course, costs. A comparison of selected features of both systems is presented in Table 1. As in the case of the unit-dose system, the cost of the multi-dose system depends, among other things, on the needs of the hospital, the number of wards, or the capacity of the machines. 

It should also be noted that in the case of both systems, it is difficult to identify specific qualitative effects. The studies cited above indicate a reduction in medication errors when using systems that support automatic drug distribution, including a reduction in the number of errors in prescribing or dispensing drugs to patients. However, the extent of these effects largely depends on the functionality of the systems and the extent of their actual use in hospitals.

Despite the many advantages of automatic drug distribution systems in hospital pharmacies, it should be noted that in order to achieve maximum effects from changing the distribution of drugs, it is necessary to ensure proper preparation of the medical entity. First of all, it is necessary to properly plan the implementation, including the preparation of rooms, new procedures, changes in the positions of many employees, and the training of medical staff. Only an appropriate organizational culture, open and facilitating the introduction of such changes, will allow for full use of the functionality of the devices, which will have maximum cost and quality effects.

## 7. Conclusions

The pharmacy is an extremely important element of the structure of each hospital, and its organization and management have a huge impact on the generally understood quality of treatment and the safety of patients staying in the hospital.

Pharmacotherapy is one of the pillars of every hospital’s activity. Activities related to drug management, from the preparation of the formula through purchase and the distribution system, translate not only into the statutory activity of the hospital but also into many other aspects related to cost and personnel policy, organization, and legal liability.

The challenges faced by health care systems, including healthcare entities, including, e.g., the increasing number of patients hospitalized with multiple diseases and thus using many drugs, not only generate much higher costs but also translate into a greater risk of making a mistake. Therefore, it is extremely important for hospital pharmacies to consider the possibility of supporting hospital drug distribution with automatic systems.

Automatic distribution of drugs in hospitals using single-dose or multi-dose systems allows for greater involvement of pharmacists in monitoring therapy and thus improves patient safety. Greater quality of care means faster health effects, shorter hospitalization time, lower exposure to the risk of medication errors, and increased quality of life and subjective well-being for patients. Automated systems also allow nurses to save time, which in turn significantly increases their involvement in direct patient care and also improves the quality of medical services. One should also take into account the area of drug costs, which can be significantly rationalized when using the devices discussed in the paper.

In conclusion, models of automatic drug distribution in the hospital show invaluable therapeutic, economic, and organizational benefits and should be the target systems in modern hospital facilities.

## Figures and Tables

**Table 1:** Comparison of multi-dose and unit-dose systems.

	Unit-Dose System	Multi-Dose System
Installation location	The system is in the hospital pharmacy, so there is a need to allocate a large space for the device.	The system is installed in hospital wards, usually replacing a classic first aid kit.
Types of supported drugs	The system mainly handles medicines in tablet form.	The system supports all medications, including syrups, ointments, and vials.
Changes of orders, emergency situations	The system, which is equipped with drug prescription software, displays interaction messages. It is possible to change medications, but at the stage of writing orders. In emergency situations, it is necessary to use a traditional first aid kit.	The system is flexible, equipped with medication ordering software, and displays interaction messages; it is possible to change orders and quickly remove the medicine from the machine in an emergency.
Cost of purchase and use	The varying costs, depending on the functionality of the device, are usually around several million euros. Electricity and service costs. Relatively high operating costs, including sachets for packaging medicines.	The costs depend on the number of departments in the hospital and the functionality of the machines. It is possible to purchase devices for only a few hospital departments.
Popularity of solutions	The system operates mainly in the United States and Japan. In Poland, it operates in several hospitals, e.g., Grudziądz, Sosnowiec, Wrocław.	The system operates, e.g., in many hospitals in Italy. In Poland, it was implemented in the hospital in Ostrołęka.

## Data Availability

All data are available from the corresponding author.
